# Vitamin D Supplementation During Neoadjuvant Chemotherapy for Breast Cancer Improves Pathological Complete Response: A Prospective Randomized Clinical Trial

**DOI:** 10.1002/wjs.12587

**Published:** 2025-04-14

**Authors:** Enver Özkurt, Cetin Ordu, Tolga Özmen, Ahmet Serkan Ilgun, Gursel Soybir, Filiz Celebi, Ertan Koç, Naziye Ak, Gul Alco, Sevgi Kurt, Filiz Ağaçayak, Ekrem Yavuz, Sıtkı Tuzlalı, Vahit Ozmen

**Affiliations:** ^1^ Department of Surgery Demiroglu Bilim University Istanbul Türkiye; ^2^ Department of Medical Oncology Gayrettepe Florence Nightingale Hospital Istanbul Türkiye; ^3^ Massachusetts General Hospital Harvard Medical School Boston Massachusetts USA; ^4^ Department of Surgery Mater Dei Hospital Birkirkara Malta; ^5^ Department of Surgery Sisli Memorial Hospital Istanbul Türkiye; ^6^ Department of Radiology Yeditepe University Medical School Istanbul Türkiye; ^7^ Istanbul Statistic Academy Istanbul Türkiye; ^8^ Department of Medical Oncology Demiroğlu Bilim University Istanbul Türkiye; ^9^ Department of Radiation Oncology Gayrettepe Florence Nightingale Hospital Istanbul Türkiye; ^10^ Department of Plastic & Reconstructive Surgery Istanbul Florence Nightingale Hospital Istanbul Türkiye; ^11^ Department of Radiology Istanbul Florence Nightingale Hospital Istanbul Türkiye; ^12^ Department of Pathology Istanbul Faculty of Medicine Istanbul University Istanbul Türkiye; ^13^ Department of Pathology Tuzlalı Pathology Istanbul Türkiye; ^14^ Department of Surgery, Head of Breast Surgery Unit Istanbul Florence Nightingale Hospital Istanbul Türkiye

**Keywords:** breast neoplasms, dietary supplements, ERBB2 protein, human, neoadjuvant systemic therapy, pathologic complete response, vitamin D

## Abstract

**Introduction:**

Achieving a pCR serves as a biomarker indicating enhanced overall survival for breast cancer patients undergoing NST. Vitamin D enhances the antitumor effect of chemotherapeutics as demonstrated in cancer cells and animal models. In this prospective randomized clinical study, we aim to investigate the effect of oral vitamin D supplementation during neoadjuvant systemic therapy (NST) on pathologic complete response (pCR).

**Methods:**

Between June 2019 and June 2023, an oral form of 50,000 IU vitamin D3 (cholecalciferol) replacement was administered once a week during NST for the study group.

**Results:**

There were 114 (50.2%) cases in the study group and 113 (49.8%) in the control group (totally 227 cases). Factors that positively influenced pCR were higher clinical T stage, higher AJCC clinical stage, Estrogen receptor negativity, progesterone receptor negativity, human epidermal growth factor receptor 2 positivity, high Ki‐67 expression (≥ 20%), hormone negative molecular subtypes, and vitamin D intake in univariate analysis. In the multivariate analysis, factors significantly affecting pCR were vitamin D intake (OR: 2.33, 95% CI 1.20–4.53; *p* = 0.013), hormone receptor negativity (OR: 2.22, 95% CI 1.11–4.43; *p* = 0.024), and Ki‐67 ≥ 20% (OR: 3.27, 95% CI 1.03–10.34; *p* = 0.044).

**Conclusions:**

This is the first and only study to compare the effect of oral vitamin D supplementation on pCR during NST. Vitamin D supplementation during NST has a significant effect on pCR in breast cancer patients. Although this effect is not significant for axillary pCR, there is an almost significant correlation.

**Trial Registration:**

ClinicalTrials.gov (Identifier: NCT03986268)

## Graphical abstract

Achieving a pCR serves as a biomarker indicating enhanced overall survival for breast cancer patients undergoing NST. Vitamin D enhances antitumor effect of chemotherapeutics as demonstrated in cancer cells and animal models. In this prospective randomized clinical study, we aim to investigate the effect of oral vitamin D supplementation during neoadjuvant systemic therapy (NST) on pathologic complete response (pCR).

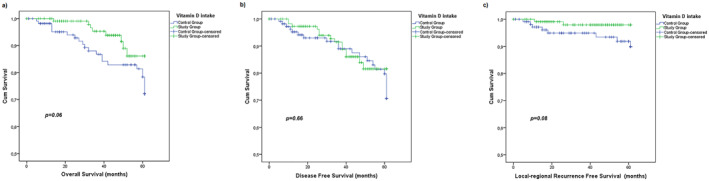

## 1 Introduction

Breast cancer is the most common cancer all around the world although it mostly affects the female population. It is also the leading cause of cancer related death in women [1]. Recently, neoadjuvant systemic therapy (NST) is adopted more and more in the setting of locally advanced breast cancer and early stage breast cancer with special subtypes like human epidermal growth factor receptor 2 positive (HER2+) and triple negative (TN) [2]. Several trials have proposed a connection between chemotherapy response and survival, a correlation further substantiated by two extensive meta‐analyses [3, 4]. Consequently, achieving a pathologic complete response (pCR) serves as a biomarker indicating enhanced overall survival (OS) [5].

Vitamin D appears to have a pivotal role in the cell cycle pathway, particularly in the context of breast cancer. Preclinical evidence suggests that vitamin D influences the control of cancer cell proliferation [6]. Furthermore, it exhibits protective properties against cancer through the induction of apoptosis, stimulation of cell differentiation, anti‐inflammatory and anti‐proliferative effects, as well as the inhibition of angiogenesis, invasion, and metastasis [6]. It is also emphasized that vitamin D enhances the antitumor effect of chemotherapeutics and drugs like doxorubicin, paclitaxel and tamoxifen causing a chemotherapy‐induced cell death in cancer cells and animal models [7, 8, 9]. The optimal impact of this effect is observed when vitamin D is administered either before or during chemotherapy [10]. Besides, some studies showed pCR is directly correlated with vitamin D levels in the setting of NST [11, 12].

In this prospective randomized clinical study enrolling breast cancer patients with NST, we aim to investigate the effect of oral vitamin D supplementation during chemotherapy on pCR. A commentary on the primary results of this prospectively collected randomized cohort study has been published previously [13] [NCT03986268].

## 2 Patients and Methods

### 2.1 Patient Cohort and Vitamin D

Female with primary breast cancer patients aged ≥ 18 years of age, who received NST between June 2019 and June 2023 in İstanbul Florence Nightingale Hospital and whose vitamin D levels were analyzed before and after NST were included in the study. Patients who consent to participate in this study and continued their follow‐up from the initiation of NST to the final surgery were randomized for the study group ‐vitamin D intake‐ (*n* = 114) and the control group ‐no vitamin D intake‐ (*n* = 113) in the order of their admission to the clinic on a 1:1 basis according to allocation described below. Primary surgeon generated the random allocation sequence, enrolled participants, and assigned participants to interventions. Patients with comorbidities that could directly or indirectly affect serum vitamin D levels, pregnant or lactating patients, and patients over 80 years of age were excluded from the study. Patient records were collected and analyzed prospectively.

An oral form of 50,000 IU vitamin D3 (cholecalciferol) replacement was administered once a week during chemotherapy for the study cohort. Serum vitamin D levels, expressed as 25‐hydroxyvitamin D (25(OH)D), before the initiation and after the final dose of NST were analyzed in the same institution using the same routinely calibrated equipment. Vitamin D levels were defined as deficient < 20 ng/mL, insufficient ≥ 20 – < 30 ng/mL, normal ≥ 30 – < 100 ng/mL, and hypervitaminosis ≥ 100 ng/mL according to the Institute of Medicine (IOM) guidelines [14].

### 2.2 Chemotherapy Regimens and Pathologic Evaluation

Neoadjuvant systemic therapy was decided by a multidisciplinary breast cancer specific tumor board according to the current international guidelines such as National Comprehensive Cancer Network (NCCN) guidelines. Standard regimens of anthracyclines and/or taxanes with or without trastuzumab and/or pertuzumab for HER2+ breast cancer cases were administered. The surgical technique was decided by the breast surgeon according to the clinical and radiological assessment of the patient before and after NST. Sentinel lymph node biopsy was performed by dual technique (blue dye and radioisotope) and at least three sentinel lymph nodes were sampled. No axillary dissection was performed if negative for metastases.

All pathological evaluations were performed by an experienced breast specific pathologist. Estrogen receptor (ER) and progesterone receptor (PgR) expression were considered positive if nuclear staining was ≥ 1%. HER2 status was assessed according to the recommendations of the American Society of Clinical Oncology/College of American Pathologists (ASCO/CAP) [15]. pCR was defined as complete disappearance of invasive carcinoma cells, including lymph nodes, regardless of the presence of residual ductal carcinoma in situ in the breast. Ki‐67 indexes were scored by counting the number of positively stained nuclei and were expressed as a percentage of total tumor cells. Ki‐67 ≥ 20% was used as a cut‐off for high proliferative indexes. The tumor subtypes were divided into the following four groups: HR+/HER2−, HR+/HER2+, HR−/HER2+ and TN (ER−, PgR− and HER2−). The American Joint Committee on Cancer (AJCC) eighth edition is used for staging [16].

### 2.3 Statistical Analysis

The sample size was determined with a 5% margin of error (95% confidence interval), 80% power, and the standard effect size was determined as 0.38. It was decided to include *n* = 110 cases in each group. In the sample allocation, in order to create homogeneous groups, patients were stratified according to their demographic characteristics and a random sampling method was used according to the order of arrival. To assess differences in categorical and continuous variables, Pearson's Chi‐square test, independent samples *t*‐test, and two‐way ANOVA tests were used. Simple linear regression and binary linear regression analyses were conducted in order to define the predictive effect of variables. Overall survival, disease‐free survival (DFS), and local‐regional recurrence free survival (LRFS) were determined by Kaplan Meier survival curves. A log rank test was used to compare survival. All *p*‐values were two‐sided and a *p*‐value of < 0.05 considered as significant within 95% confidence interval (CI). All statistical analyses were performed using the SPSS version 23.0 (IBM Inc., Armonk, USA). The data was analyzed by Enver Özkurt (MD., Assoc, Prof.) and Ertan Koç (statistician).

The research protocol was approved by relevant institutional review boards or ethics committees and that all human participants gave written informed consent. This study was performed in line with the principles of the Declaration of Helsinki.

## 3 Results

A total of 237 eligible patients were prospectively enrolled. Ten patients discontinued follow‐up and 227 patients were included in the final analysis. Patient demographics and clinicopathological characteristics are summarized in Tables 1 and 2. There were 114 (50.2%) cases in the study group and 113 (49.8%) in the control group. When compared, there was no statistically significant difference between the clinicopathological characteristics of the study and control groups, except for primary biopsy histology, PgR expression, type of breast and axillary surgery, and 25(OH)D levels after NST (Table 1). Patients in the study group has undergone conservative treatments more than the ones in the control group (Lumpectomy, 64.8% vs. 46%, *p* = 0.001 and sentinel lymph node biopsy, 63.2% vs. 50.5%, *p* = 0.008 respectively). Factors that positively influenced pCR were higher clinical T stage, higher AJCC clinical stage, ER negativity, PgR negativity, HER2 positivity, high Ki‐67 expression (≥ 20%), hormone negative molecular subtypes (HER2 overexpression and triple negative) and finally vitamin D intake in univariate analysis (Table 2).

**TABLE 1:** Patient demographics and clinicopathological characteristics for control and study groups.

	Total (*n* = 227)	Control group *n* = 113 (49.8%)	Study group *n* = 114 (50.2%)	*p*‐value
Age	24–74			
Median	46	46 (24–74)	47.5 (27–73)	0.852
Menopausal status				
Premenopausal	140 (61.7%)	68 (60.2%)	72 (63.2%)	0.644
Postmenopausal	87 (38.3%)	45 (39.8%)	42 (36.8%)	
Family history of breast cancer (*n* = 209)				
No	133 (63.6%)	63 (63%)	70 (64.2%)	0.855
Yes	76 (36.4%)	37 (37%)	39 (35.8%)	
Co‐morbidities				
No	189 (83.3%)	89 (78.8%)	100 (87.7%)	0.071
Yes	38 (16.7%)	24 (21.2%)	14 (12.3%)	
Clinical T stage				
cT1	76 (33.5%)	36 (31.9%)	40 (35.1%)	0.361
cT2	116 (51.1%)	58 (51.3%)	58 (50.9%)	
cT3	32 (14.1%)	16 (14.2%)	16 (14.0%)	
cT4	3 (1.3%)	3 (2.7%)	0 (0%)	
Clinical N stage				
cN−	30 (13.2%)	19 (16.8%)	11 (9.6%)	0.111
cN+	197 (86.8%)	94 (83.2%)	103 (90.4%)	
AJCC 8 ‐ clinical stage				
Stage 1	16 (7%)	8 (7.1%)	8 (7%)	0.976
Stage 2	178 (78.4%)	88 (77.9%)	90 (78.9%)	
Stage 3	33 (14.5%)	17 (15.0%)	16 (14.0%)	
Tumor focality				
Unifocal	190 (83.7%)	91 (80.5%)	99 (86.8%)	0.437
Multifocal/centric	37 (16.3%)	21 (19.5%)	15 (13.2%)	
Biopsy result^a^				
IDC	195 (85.9%)	89 (78.8%)	106 (93.0%)	**0.007**
ILC	16 (7.0%)	11 (9.7%)	5 (4.4%)	
Other	16 (7.0%)	13 (11.5%)	3 (2.6%)	
Histologic grade^a^				
Grade 1	3 (1.3%)	3 (2.7%)	0 (0)	0.097
Grade 2	93 (41.0%)	41 (36.3%)	52 (45.6%)	
Grade 3	131 (57.7%)	69 (61.1%)	62 (54.4%)	
ER^a^				
Positive	155 (68.3%)	74 (65.5%)	81 (71.1%)	0.368
Negative	72 (31.7%)	39 (34.5%)	33 (28.9%)	
PR^a^				
Positive	125 (55.1%)	53 (46.9%)	72 (63.2%)	**0.014**
Negative	102 (44.9%)	60 (53.1%)	42 (36.8%)	
HER2/neu^a^				
Positive	82 (36.1%)	34 (30.1%)	48 (42.1%)	0.059
Negative	145 (63.9%)	79 (69.9%)	66 (57.9%)	
Ki‐67 (%) (*n* = 202)^a^	4–95			
Mean	39.9	39.4	40.3	0.793
Median	35	34	35	
Subgroups^a^				
HR (+)/HER2 (−)	106 (46.7%)	56 (49.6%)	50 (43.9%)	0.155
HR (+)/HER2 (+)	55 (24.2%)	21 (18.6%)	34 (29.8%)	
HR (−)/HER2 (+)	28 (12.3%)	13 (11.5%)	15 (13.2%)	
TN	38 (16.7%)	23 (20.4%)	15 (13.2%)	
Subgroups (Dichotomized)^a^				
Hormone only (+)	94 (44.8%)	46 (45.1%)	48 (44.4%)	0.924
Other (TN and HER2 positive)	116 (55.2%)	56 (54.9%)	60 (55.6%)	
Vitamin‐D level before NST				
Mean	23.38 ± 8.96	23.54 ± 8.80	23.39 ± 9.31	0.9
Vitamin‐D group (before NST)				
Deficient (< 20 ng/mL)	98 (43.2%)	48 (42.5%)	50 (43.8%)	0.942
Insufficient (20–30 ng/mL)	71 (31.3%)	35 (31.0%)	36 (31.6%)	
Normal (30–100 ng/mL)	58 (25.6%)	30 (26.5%)	28 (24.6%)	
Vitamin‐D group (after NST)^b^				
Deficient (< 20 ng/mL)	57 (26.3%)	57 (55.3%)	0	**< 0.001**
Insufficient (20–30 ng/mL)	27 (12.4%)	27 (26.2%)	0	
Normal (30–100 ng/mL)	124 (57.1%)	19 (18.4%)	111 (97.4%)	
Hypervitaminosis (> 100 ng/mL)	9 (4.9%)	0	3 (2.6%)	
Type of breast surgery				
Mastectomy	101 (44.5%)	61 (64%)	40 (35.2%)	**< 0.001**
Lumpectomy	126 (55.5%)	52 (46.0%)	74 (64.8%)	
Axillary surgery				
SLNB	129 (56.8%)	57 (50.5%)	72 (63.2%)	**0.008**
ALND	42 (18.5%)	30 (26.5%)	12 (10.5%)	
SLNB + ALND	56 (24.7%)	26 (23.0%)	30 (26.3%)	
Follow‐up time				
Mean (± STD)	8–102			
	41.4 (± 18.9)	43.1 (± 21.4)	39.6 (± 15.9)	0.165

*Note:* Bold values are the statistically significant factors.

Abbreviations: AJCC, American Joint Committee on Cancer; ALND, axillary lymph node dissection; ER, estrogen receptor; IDC, invasive ductal carcinoma; ILC, invasive lobular carcinoma; HER2/neu, human epidermal growth factor receptor 2; HR, hormone receptor; MRM, modified radical mastectomy; NST, neoadjuvant systemic therapy; PR, progesterone receptor; SLNB, sentinel lymph node biopsy; TN, triple negative.

^a^ According to pre‐neoadjuvant systemic therapy biopsy results.

^b^ *n* = 103 for the control group.

**TABLE 2:** Patient demographics and clinicopathological characteristics for pathologic complete response and no pathologic complete response groups.

	No‐pCR *n* = 163 (71.8%)	pCR *n* = 64 (28.2%)	*p*‐value
Menopausal status			
Premenopausal	100 (61.3%)	40 (62.5%)	0.873
Postmenopausal	63 (38.7%)	24 (37.5%)	
Clinical T stage			
cT1	53 (32.5%)	23 (35.9%)	**0.014**
cT2	77 (47.2%)	39 (60.9%)	
cT3	30 (18.4%)	2 (3.1%)	
cT4	3 (1.8%)	0 (0%)	
Clinical N stage			
cN−	25 (15.3%)	5 (7.8%)	0.132
cN+	138 (84.7%)	59 (92.2%)	
AJCC 8 ‐ clinical stage			
Stage 1	12 (7.4%)	4 (6.3%)	**0.008**
Stage 2	120 (73.6%)	58 (90.6%)	
Stage 3	31 (19%)	2 (3.1%)	
Biopsy result^a^			
IDC	114 (88.3%)	51 (79.7%)	0.120
ILC	11 (6.7%)	5 (7.8%)	
Other	8 (4.9%)	8 (12.5%)	
Histologic grade^a^			
Grade 1	3 (1.8%)	0 (0%)	0.408
Grade 2	69 (42.3%)	24 (37.5%)	
Grade 3	91 (55.9%)	40 (62.5%)	
ER^a^			
Positive	123 (75.5%)	32 (50.0%)	**< 0.001**
Negative	40 (24.5%)	32 (50.0%)	
PR^a^			
Positive	99 (60.7%)	26 (40.6%)	**0.006**
Negative	64 (39.3%)	38 (59.4%)	
HER2/neu^a^			
Positive	51 (31.3%)	31 (48.4%)	**0.016**
Negative	112 (68.7%)	33 (51.6%)	
Ki‐67 (%) (*n* = 202)^a^			
Mean	32 (22.5%)	4 (6.7%)	**0.007**
Median	110 (77.5%)	56 (93.3%)	
Subgroups^a^			
HR (+)/HER2 (−)	87 (53.4%)	19 (29.7%)	**0.001**
HR (+)/HER2 (+)	39 (23.9%)	16 (25.0%)	
HR (−)/HER2 (+)	13 (8.0%)	15 (23.4%)	
TN	24 (14.7%)	14 (21.9%)	
Subgroups (Dichotomized)^a^			
Hormone only (+)	86 (52.8%)	19 (29.7%)	**0.002**
Other (TN and HER2 positive)	77 (47.2%)	45 (70.3%)	
Vitamin D intake			
No	94 (57.7%)	19 (29.7%)	**< 0.001**
Yes	69 (42.3%)	45 (70.3%)	

*Note:* Bold values are the statistically significant factors.

Abbreviations: AJCC, American Joint Committee on Cancer; ER, estrogen receptor; HER2/neu, human epidermal growth factor receptor 2; HR, hormone receptor; IDC, invasive ductal carcinoma; ILC, invasive lobular carcinoma; pCR, pathologic complete response; PR, progesterone receptor; TN, triple negative.

^a^ According to pre‐neoadjuvant systemic therapy biopsy results.

Pathological complete response associated with vitamin D supplementation is evaluated in Table 3. Although pCR was defined as no residual tumor in the breast and axilla, we also aimed to determine the effect of vitamin D on axillary pCR alone. For this purpose, only initially clinical node‐positive cases (*n* = 197) were included in the analysis of axillary pCR (Table 3). Of 197 patients with positive clinical nodes, 186 were diagnosed with FNA and 9 with positron emission tomography. Vitamin D intake had a significant effect on pCR, but not for the axilla. Nevertheless, there was a trend in favor of achieving axillary pCR (Table 3).

**TABLE 3:** Pathologic complete response rates in the control and study groups for breast, axilla, and all cases.

Pathologic complete response	Control group *n* = 113 (49.8%)	Study group *n* = 114 (50.2%)	*p*‐value
Breast pCR (*n* = 227)			
pCR	19 (16.8%)	45 (39.5%)	**< 0.001**
No‐pCR	94 (83.2%)	69 (60.5%)	
Axilla pCR (*n* = 197)			
pCR	29 (30.9%)	44 (42.7%)	0.085
No‐pCR	65 (69.1%)	59 (52.3%)	
Breast + axilla pCR (*n* = 197)			
pCR	10 (10.6%)	25 (24.3%)	**0.012**
No‐pCR	84 (89.4%)	78 (75.7%)	

*Note:* Bold values are the statistically significant factors.

Abbreviation: pCR, pathologic complete response.

Vitamin D levels were significantly high in the study group after NST compared to initial levels (mean 71.27 ± 16.76 SD vs. 23.29 ± 9.31 SD; *p* < 0.001). On the other hand, it was significantly low in the control group before and after NST (mean 23.54 ± 8.80 SD vs. 22.37 ± 8.62 SD; *p* < 0.001). There was no statistical difference between the pCR rates of the initially deficient/insufficient vitamin D group and the normal vitamin D group (27.8% and 29.3%, respectively; *p* = 0.827). In the multivariate analysis, factors significantly affecting pCR were vitamin D intake (OR: 2.33, 95% CI 1.20–4.53; *p* = 0.013), hormone receptor negativity (OR: 2.22, 95% CI 1.11–4.43; *p* = 0.024), and Ki‐67 ≥ 20% (OR: 3.27, 95% CI 1.03–10.34; *p* = 0.044) (Table 4). Vitamin D intake was the most effective factor of all as the analysis was conducted by forward LR (logistic regression) method (Table 4).

**TABLE 4:** Multivariate analysis of factors affecting pathologic complete response.

	OR	95% CI		*p*‐value
		Lower	Upper	
Vitamin D intake *(ref. = Control group)*	2.329	1.199	4.526	**0.013**
Subgroups (dichotomized) *(ref. = HR + only)*	2.218	1.111	4.428	**0.024**
Clinical T stage *(ref. = cT1)*	0.963	0.478	1.938	0.915
AJCC 8 ‐ clinical stage *(ref. = Stage 1)*	0.512	0.177	1.480	0.216
Ki‐67 (< 20% vs. ≥ 20%) *(ref. = Ki‐67 < 20%)*	3.268	1.033	10.336	**0.044**

*Note:* Bold values are the statistically significant factors.

Abbreviations: AJCC, American Joint Committee on Cancer; CI, confidence interval; HR, hormone receptor; OR, odds ratio; ref., reference group.

In the subgroup analysis, vitamin D supplementation significantly affected pCR rates in patients with high Ki‐67 (≥ 20%) (*p* = 0.012) but not in patients with low Ki‐67 (*p* = 0.406). When comparing hormone‐positive and other groups (HER2‐enriched and triple‐negative), vitamin D supplementation significantly affected pCR rates in both subgroups (*p* = 0.016 and *p* = 0.004, respectively). For the clinical T stage subgroups, a significant effect was observed in both the cT1 and cT2 subgroups (*p* = 0.014 and *p* = 0.011, respectively). Finally, in the subgroup analysis according to the AJCC clinical stage, vitamin D supplementation showed a significant effect on pCR for stage II cases (*p* = 0.001), but did not reach a significant level for stage I and stage III due to the small number of cases in these groups.

**FIGURE 1:**
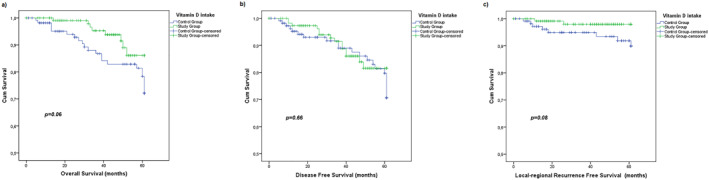
5‐Year overall survival (a), disease free survival (b), and Local‐regional recurrence free survival (c) of study and control groups.

The 5‐year OS, DFS and LRFS rates were 82%, 79.5% and 94.5%, respectively, for the entire cohort. The effect of vitamin D supplementation on survival was also analyzed between the study and control groups. The 5‐year OS rate was 86.1% versus 78.4% (*p* = 0.06); the DFS rate was 81.6% versus 79.1 (*p* = 0.66); and the LRFS rate was 98% versus 91.8 (*p* = 0.08) for the study and control groups, respectively (Figure 1).

High‐dose vitamin D supplementation can lead to several adverse effects, primarily due to hypercalcemia, which may cause symptoms such as nausea, vomiting, weakness, and kidney dysfunction. Prolonged excessive intake has been linked to vascular and soft tissue calcification, increasing the risk of cardiovascular events. There is also evidence indicating potential nephrotoxicity, as excessive vitamin D can lead to kidney stone formation and impaired renal function. Furthermore, a high‐dose of vitamin D may increase the risk of falls and fractures in older adults, possibly due to dysregulated calcium metabolism [17]. There were no harms or unintended effects in each group.

## 4 Discussion

In this prospectively designed randomized controlled study enrolling breast cancer patients with NST, we demonstrated that weekly high‐dose (50,000) vitamin D supplementation during chemotherapy significantly improved pCR rate. As its unique design, this is the first study comparing the effect of oral vitamin D intake on pCR during NST. A commentary on the primary results of this prospectively collected randomized controlled study has been published previously [13].

The effect of vitamin D on breast cancer has always been an area of interest [13, 18]. There are in vivo experiments on different mouse models showing tumor regression in response to calcitriol and vitamin D [19, 20, 21]. Jeong et al. examined the effect of dietary vitamin D and calcitriol on mouse breast tumor initiating cells [19]. They indicated that vitamin D compounds target the breast tumor initiating cells and reducing the tumor initiating activity. They also demonstrated that vitamin D compounds (vitamin D dietary supplements and calcitriol) inhibit tumor growth and that vitamin D deficiency accelerates tumor growth. Laboratory studies demonstrating that vitamin D sensitizes breast cancer cells to chemotherapy agents. Vitamin D has demonstrated the ability to enhance programmed cell death triggered by adriamycin, paclitaxel, tamoxifen, and radiotherapy [22]. In our cohort, standard regimens of anthracyclines and/or taxanes were commonly given for breast cancer cases, as recommended by recent guidelines.

Some studies have showen a significant relationship between vitamin D deficiency and breast cancer [23, 24, 25, 26] and some have not [27, 28]. In a meta‐analysis by Voutsadakis reviewing 25 studies including 8798 patients with and without breast cancer, 45.28% of patients had vitamin D deficiency (25(OH)D < 20 ng/mL) whereas it was 33.71% for control group without breast cancer [23]. For the 25(OH)D < 30 ng/mL subgroup (insufficient and deficient), it was 67.44% and 53.66 respectively. In another study about the association of serum 25(OH)D concentration with breast cancer risk in postmenopausal women in the United States [24], they presented a significant, nonlinear, invert ‘U’ relationship between serum vitamin D levels and breast cancer (*p* = 0.029). Finally, in a systematic review and meta‐analysis Hossain et al. [25] pooled the findings of 22 studies and they showed a direct association between vitamin D deficiency and breast cancer (RR pooled = 1.91, 95% CI: 1.51–2.41, *p* < 0.001). They also put forward that vitamin D intake (RR pooled = 0.99, 95% CI: 0.97–1.00, *p* = 0.022, per 100 IU/d) and supplemental vitamin D (RR pooled = 0.97, 95% CI:0.95–1.00, *p* = 0.026) were inversely associated with breast cancer [25]. As we studied the breast cancer patients in our cohort, the baseline vitamin D levels were similar for study group and control group (*p* = 0.94) (Table 1).

Neoadjuvant systemic therapy facilitates real‐time monitoring of treatment efficacy in vivo for physicians. Furthermore, it serves as a surrogate clinical endpoint for long‐term survival, particularly when a pathologic complete response (pCR) is attained [2]. Therefore, the effect of vitamin D levels on pCR has also been a subject of research. In the NEOZOTAC trial [29], they found no statistically significant pCR effect between baseline (*p* = 0.92, OR: 1.00, 95% C.I. 0.97–1.03 for pCR) and end of NST vitamin D levels (*p* = 0.80). However, there was a significant correlation between positive changes in vitamin D levels and achieving a pathological good response, indicating a more favorable outcome in patients who experience an increase or maintain stable vitamin D levels, as opposed to those with a decrease in vitamin D levels in univariate (*p* = 0.01) and multivariate analysis (*p* = 0.003) [29]. Similarly, in the I‐SPY trial [12], having sufficient levels of vitamin D was linked to higher odds of pCR compared to individuals with insufficient vitamin D levels, although this association did not reach statistical significance (*p* = 0.25, OR: 1.54; 95% CI, 0.49–4.80).

Viala et al. investigated the impact of vitamin D on pCR and survival following neoadjuvant chemotherapy for breast among 327 women [11]. In the logistic regression analysis, pCR was significantly related with higher vitamin D levels (*p* = 0.04). In the multivariate analysis, this effect stands significant. In addition, their analysis showed a near‐significant correlation between vitamin D levels and progression free survival rates in the HR+/HER2− subgroup.

In this study, we demonstrated a significant relationship between vitamin D supplementation and pCR in the univariate (*p* < 0.001) and multivariate analysis (*p* = 0.013) (Tables 3 and 4). We also performed a subgroup analysis about the effect of vitamin D intake on pCR for initially positive lymph nodes (*n* = 197). Although it is not statistically significant, there was a trend for achieving pCR on lymph nodes for patients who received vitamin D supplementation compared to those who did not (42.7% vs. 30.9% respectively; *p* = 0.085). It is also analyzed that vitamin D supplementation during NST improves the OS (86.1% vs. 78.4%; *p* = 0.06) and LRFS (98% vs. 91.8%; *p* = 0.08) although it is not significant at the *p* = 0.05 level. This non‐significance may be related to the shorter median follow‐up time and may be related to the small number of cohorts. As two thirds of the cases are hormone positive, it is likely that the significant effect would be seen in the survival analysis with longer follow‐up. Nevertheless, the present analysis shows that there is an important trend for improved survival with vitamin‐D supplementation. Additionally, patients in the study group have undergone conservative treatments more than the ones in the study group (Lumpectomy rates of 64.8% vs. 46%, *p* = 0.001 and sentinel lymph node biopsy rates of 63.2% vs. 50.5%, *p* = 0.008 respectively). Thus, it can be interpreted that vitamin D supplementation may have a positive effect on conservative surgery rates after NST.

The limitation of this study is its single‐centered design and allocation of patients by the primary surgeon. But we believe that this is in the nature of the single center trials. We try to overcome this potential bias by allocating patients in the order of their admission to the clinic on a 1:1 basis with the supervision of the biostatistician. As this is the first report on the relationship between vitamin D intake and pCR after NST, a prospective randomized multicentre trial is being considered. Another limitation is the lack of information on the optimal dose of vitamin D to achieve pCR. However, it is worth noting that there is no definitive information on this in the recent literature. Also, longer follow‐up periods may provide more definitive conclusions about the long‐term benefits of vitamin D supplementation. In addition, the cost of an additional therapeutic agent (vitamin D supplementation) and the pre‐ and post‐treatment laboratory costs to determine vitamin D levels during the trial were not reported. Finally, monitoring vitamin D levels, for example once a month, could be considered to individualize treatment and keep patients' vitamin D levels within a predetermined range.

In conclusion, this is the first and only study to compare the effect of oral vitamin D supplementation on pCR during NST. Vitamin D supplementation during NST has a significant effect on pCR in breast cancer patients. Although this effect is not significant for axillary pCR, there is an almost significant correlation. We also reported that there is a trend toward better OS and LRFS with the supplementation of vitamin D. To summarize, we believe that the addition of vitamin D should be considered for breast cancer patients before and during NST for better outcomes. But before drawing a robust conclusion the effect of vitamin D supplementation on pCR from the initiation of NST needs to be further investigated with a larger series of multicentre randomized controlled trials.

## Author Contributions

**Enver Özkurt:** conceptualization, writing – review and editing. **Cetin Ordu:** conceptualization, validation, writing – review and editing. **Tolga Özmen:** writing – review and editing. **Ahmet Serkan Ilgun:** data curation, writing – review and editing. **Gursel Soybir:** writing – review and editing. **Filiz Celebi:** writing – review and editing. **Ertan Koç:** formal analysis, methodology, software, validation. **Naziye Ak:** writing – review and editing. **Gul Alco:** writing – review and editing. **Sevgi Kurt:** writing – review and editing. **Filiz Ağaçayak:** writing – review and editing. **Ekrem Yavuz:** writing – review and editing. **Sıtkı Tuzlalı**: writing – review and editing. **Vahit Ozmen:** conceptualization, supervision, writing – review and editing.

## Ethics Statement

The research protocol was approved by relevant institutional review boards or ethics committees and that all human participants gave written informed consent. This study was performed in line with the principles of the Declaration of Helsinki.

## Conflicts of Interest

The authors declare no conflicts of interest.

## Data Availability

The datasets generated and analyzed during the current study are not publicly available because individual privacy could be compromised, but they are available from the corresponding author upon reasonable request.
